# Processed carp scales as a functional food for sepsis protection: enhancing amino acid content and bioactive peptide profiles

**DOI:** 10.3389/fnut.2026.1723040

**Published:** 2026-01-26

**Authors:** Lisha Yan, Qiong Wang, Min Tang

**Affiliations:** 1 Department of Clinical Medicine, Yiyang Medical College, Yiyang, China; 2 Department of Pharmacy, Yiyang Medical College, Yiyang, China

**Keywords:** amino acid content, anti-inflammatory peptides, bioactive peptide, carp scales, functional food

## Abstract

Carp scales represent an abundant and protein-rich by-product of aquaculture that is commonly discarded as waste. Despite their potential as a sustainable source of dietary protein, their rigid structural matrix restricts nutrient bioavailability. Moreover, sepsis-induced multiorgan injury remains a life-threatening clinical condition with high mortality, underscoring the need for novel functional nutritional strategies. Although traditional processing methods are known to alter physical properties, their effects on the nutritional composition and anti-inflammatory activity of carp scales remain poorly understood. This study aims to characterize the compositional changes induced by thermal processing and to evaluate the enhanced protective efficacy of processed versus raw carp scales in a sepsis model. Processed carp scales were prepared using optimized sand stir-frying (150 °C, 2.5 min) and evaluated through comparative analyses integrating LPS-induced sepsis models and *in vivo* experiments. Results demonstrated that thermal processing induced significant biochemical transformations, markedly improving the nutritional profile by increasing total protein content and amino acid availability. Notably, LC–MS analysis identified 10 novel bioactive peptides (e.g., PGSPGPAGPAGARGQQ) uniquely generated during heating. Pharmacologically, processed carp scales exhibited superior therapeutic effects compared to raw scales. *In vivo*, treatment with processed carp scales significantly suppressed pro-inflammatory cytokines (TNF-*α*, IL-6, IL-1β), effectively attenuated multiple organ dysfunction—evidenced by reduced ALT, AST, and BUN levels—and normalized coagulation parameters while alleviating histopathological damage. This study reveals a “processing–nutrient–efficacy” axis, demonstrating that thermal processing generates bioactive peptides that may contribute to enhanced anti-sepsis activity. These findings support the potential of processed carp scales as a promising high-value functional food ingredient.

## 1 Introduction

The common carp (*Cyprinus carpio* Linnaeus), a member of the family *Cyprinidae*, is one of the most historically cultivated and widely distributed freshwater fish species in Asia. In China, it represents a cornerstone of the aquaculture industry due to its high yield and economic significance. However, the rapid expansion of large-scale farming and processing has led to the generation of substantial by-products, which account for 30–50% of the total fish mass. Among these, fish scales constitute a major portion. Despite being rich in organic components, fish scales are frequently discarded as waste, causing significant environmental pollution and resource inefficiency (1, 2). Structurally, fish scales are bio-composites consisting of type I collagen fibers highly mineralized with hydroxyapatite, resulting in a rigid and insoluble matrix. This unique physical characteristic limits the direct release of bioactive nutrients and results in poor bioavailability in their raw form, thereby hindering their application in the food and pharmaceutical industries. Therefore, developing effective processing strategies to disrupt this matrix and release high-value bioactive components has become a prominent research focus.

Chemically, proteins account for approximately half of the fish scale’s mass, making them an excellent source of collagen and keratin. Current development in high-value utilization primarily focuses on preparing bioactive peptides through enzymatic hydrolysis. Previous studies have demonstrated that fish scale peptides exhibit functional activities, such as antioxidant, anti-ACE (angiotensin-converting enzyme), metal ion chelation, and immunomodulatory properties (3). To date, anti-inflammatory peptides have been extracted from catfish (4), tilapia (5, 6), *Hypophthalmichthys molitrix* (7), lanternfish (8), and anglerfish (9). However, existing extraction methods often involve complex chemical procedures or expensive enzymes, limiting industrial scalability. Furthermore, while traditional thermal processing (such as stir-frying) is a classic method to modify the properties of medicinal materials, its specific impact on the changes in nutritional components and peptide generation of carp scales remains largely unexplored. There is a lack of systematic research comparing how processing transforms the inert raw scales into bioactive functional ingredients.

The exploration of anti-inflammatory peptides is particularly relevant to critical care medicine. Sepsis is a life-threatening organ dysfunction syndrome triggered by a dysregulated host response to infection (10). During sepsis, the immune system releases an uncontrolled storm of inflammatory mediators (e.g., TNF-*α*, IL-6), which, coupled with coagulation abnormalities, leads to multi-organ damage (liver, kidneys, lungs) (11). Although current therapeutic strategies focus on antibiotics and organ support, the high mortality rate necessitates novel adjunctive therapies. Bioactive peptides derived from natural food sources have gained increasing attention due to their high safety profile and dual function of nutritional support and immune regulation (12). Consequently, transforming carp scales into an effective source of anti-inflammatory peptides offers a promising strategy for sepsis management.

To address the challenges of low bioavailability of raw carp scales and the need for natural anti-inflammatory active ingredients, this study aims to systematically evaluate the effects of traditional processing methods (sand stir-frying) on the chemical composition and anti-inflammatory activity of carp scales. Unlike previous studies that solely focus on enzymatic hydrolysis, the specific objectives of this study are: (1) to systematically investigate the compositional transformation between raw and processed carp scales; (2) to identify novel small-molecule peptides generated during processing using LC–MS; and (3) to validate the enhanced anti-inflammatory and organ-protective efficacy of processed scales in an *in vivo* sepsis model. This study seeks to provide a scientific foundation for converting this underutilized aquaculture by-product into high-value functional foods with the potential to prevent inflammation-related diseases.

## 2 Materials and methods

### 2.1 Materials and reagents

#### 2.1.1 Preparation of carp scale samples

Carp scales were sourced from an aquatic market in Yiyang, Hunan Province, China. Fresh scales were collected and stored at −80 °C for subsequent use. For the preparation of raw carp scale samples (Cc-LI), the scales were first dried. Subsequently, 10 g of dried scales were placed in a 250 mL round-bottom flask, followed by the addition of 100 mL of purified water. The mixture was refluxed in a water bath at 100 °C for 1 h. After filtration, the resulting filtrate was freeze-dried to obtain a solid powder. For the preparation of processed carp scale samples (Cc-LII), 500 g of river sand was heated to 150 °C, and 20 g of raw carp scales were added and stir-fried for 2.5 min. Subsequently, 10 g of the stir-fried scales were transferred to a 250 mL round-bottom flask, and 100 mL of purified water was added. The mixture was refluxed in a water bath at 100 °C for 1 h, then filtered, and the filtrate was freeze-dried to obtain a solid powder. Raw carp scales were extracted at 30.9% and processed carp scales at 45.4%. The screening process for samples (1–7) is detailed in Supplementary Table S1 and Supplementary Figure S1.

#### 2.1.2 Instruments

The analysis was performed using a Waters Acquity Ultra-Performance Liquid Chromatography (UPLC) system equipped with a four-element pump and TUV detector, coupled with a Waters Xevo G2 Q-TOF mass spectrometry system (Waters, Milford, MA, United States). Chromatographic separation was carried out on an ACQUITY UPLC BEH C18 column (2.1 mm × 50 mm, 1.7 μm, Waters, United States). Additionally, the study utilized an MS205DU electronic analytical balance (Mettler Toledo, Switzerland), a KQ5200 ultrasonic cleaner (Kunshan Ultrasonic Instruments Co., Ltd., China), and a PURELAB Option-R7 water purification system (ELGA, United Kingdom). Optical density (OD) was measured using a SpectraMax i3x multi-mode microplate reader (Molecular Devices, San Jose, CA, United States). Easy-nLC 1,200/QExactive Liquid chromatography-mass spectrometer (Thermo Fisher Scientific, United States) and Analytical column: 150 μm i.d. × 170 mm, packing: Reprosil-Pur 120 C18-AQ 1.9 μm (2.1 mm × 50 mm, 1.7 μm, Thermo Fisher Scientific, United States) for protein mass spectrometry analysis. For pathological analysis, tissue sections were imaged using an optical microscope (BX53, Olympus, Tokyo, Japan). Serum biochemical indices were analyzed using an automatic biochemical analyzer (7,020, Hitachi, Tokyo, Japan), and coagulation parameters were determined using an automated coagulation analyzer (CA-500, Sysmex, Kobe, Japan).

#### 2.1.3 Reagents

RAW264.7 macrophages were purchased from Procell Life Science and Technology Co., Ltd. (Wuhan, China). Lipopolysaccharide (LPS), derived from *Escherichia coli* O11: B4, was sourced from Sigma-Aldrich (St. Louis, Missouri, United States). ELISA kits for TNF-*α* (Catalog No.: JL13202), IL-1β (Catalog No.: JL20884), IL-6 (Catalog No.: JL20896), and HMGB-1 (Catalog No.: JL13892) were acquired from Jianglai Biotechnology (Shanghai, China). Formic acid was sourced from CNW Technologies GmbH (Germany), acetonitrile (HPLC grade) from Merck KGaA (Germany), and NH₄HCO₃ (analytical grade) from Sinopharm Chemical Reagent Co., Ltd. (China).

### 2.2 Analysis of the composition of carp scales

#### 2.2.1 Determination of amino acid content

Approximately 1 g of the sample was weighed and mixed with 10 mL of hydrochloric acid. The mixture was purged with nitrogen gas and hydrolyzed at 110 °C for 22 h. After cooling to 25 °C, the solution was diluted to 50 mL with distilled water, further diluted 1,000-fold, and filtered before instrumental analysis. A mixed standard solution containing 17 amino acids was prepared, and a corresponding calibration curve was generated utilizing distilled water. Liquid chromatography conditions were as follows: column: XB-C18 reversed-phase column (2.1 × 100 mm, 2.6 μm); mobile phase: A: 5 mmol/L ammonium acetate; B: methanol; gradient elution: 0–2 min, A = 85%; 2–5 min, A decreased to 70%; 5–8 min, A = 10%; 8–11 min, A increased to 85%; flow rate: 0.5 mL/min; column temperature: 30 °C; injection volume: 10 μL. Mass spectrometry conditions were as follows: ionization source: electrospray ionization (ESI); curtain gas (psi): 35; ionization voltage (V): 5,500 (positive ion mode); temperature (°C): 550; nebulizer gas (psi): 55; auxiliary heating gas (psi): 55; scanning mode: multiple reaction monitoring.

#### 2.2.2 Determination of protein content

The total protein content was determined using the classic Kjeldahl method (AOAC Official Method 984.13). Briefly, the sample was weighed and placed into a digestion tube, followed by the addition of copper sulfate, potassium sulfate, and sulfuric acid to facilitate digestion. The process continued until the liquid turned clear green. The digested sample was then distilled using a Kjeldahl apparatus, and the distillate was absorbed in a boric acid solution. A standard hydrochloric acid solution was used for titration, and the nitrogen content was calculated based on the volume of acid consumed. The protein content was subsequently determined by multiplying the nitrogen content by a conversion factor. The nitrogen content was calculated based on the volume of standard hydrochloric acid consumed during titration. Finally, the total protein content was calculated by multiplying the nitrogen content by a conversion factor of 6.25.

#### 2.2.3 Protein mass spectrometry analysis

Freeze-dried powder (5 mg) from both raw and processed carp scales was separately dissolved in 5 mL of 1% NH₄HCO₃ solution, filtered through a 0.22 μm microporous membrane, and used as test samples. Liquid chromatography conditions were as follows: mobile phase A: 0.1% formic acid in water, B: acetonitrile, with gradient elution (0–25 min, 5% B → 20% B; 25–40 min, 20% B → 50% B; 40–41 min, 50% B → 99% B; 41–42 min, 99% B; 42–43 min, 99% B → 5% B; 43–45 min, 5% B); flow rate: 0.3 mL/min; column temperature: 40 °C; injection volume: 1 μL. Mass spectrometry conditions were as follows: ionization mode: ESI+; capillary voltage: 3 kV; cone voltage: 40 V; desolvation temperature: 450 °C; desolvation gas flow: 600 L/h; ion source temperature: 120 °C. Data acquisition was performed using MSE and MS/MS modes with an acquisition time of 45 min, a scan range of 100–1,500 amu, a scan time of 0.2 s, and a cone voltage of 40 V. In MSE mode, the collision energy was 4 V for low energy and ramped from 20 to 30 V for high energy; collision energy in MS/MS mode was 25 V. High-purity argon served as the collision gas.

The original mass spectrum files were analyzed using PEAKS *De novo* method for peptide sequence analysis, and the retrieval parameters were as follows:

1. Fixed modifications: Carbamidomethyl (C).
2. Variable modifications: Oxidation (M), Acetyl (Peptide N-term).
3. Enzyme: Non specific.
4. Peptide Mass Tolerance: 20 ppm.
5. Fragment Mass Tolerance: 0.02 Da.

### 2.3 Screening of anti-inflammatory activity *in vitro*

RAW264.7 cells in the logarithmic growth phase were harvested using trypsin and seeded into 96-well plates at a density of 1 × 10^4^ cells per well (13). The cells were cultured in a 5% CO₂ incubator at 37 °C for 24 h to allow for adhesion. After discarding the supernatant, the cells were assigned into the following groups, each with six replicates: control group, 100 μL of blank medium; model group, 100 μL of LPS (1 μg/mL); positive control group, 100 μL of dexamethasone (10 μg/mL); and treatment groups (the drug dose was 0, 1, 2, 3, 4, 5 mg/mL), 100 μL of drug-containing medium at different concentrations. Following treatment, the plates were incubated under the same conditions for an additional 24 h. After 24 h, 10 μL of CCK-8 solution was added to each well, and the plates were further incubated for 2 h. Absorbance was then measured at 450 nm.

RAW264.7 cells were seeded into 24-well plates at a density of 3 × 10^5^ cells per well, with three replicates per group, and cultured in a 5% CO₂ incubator at 37 °C for 24 h. The resulting supernatant was then removed, and the cells were assigned into the following groups: control group, 500 μL of the blank medium; model group, 500 μL of LPS at 1 μg/mL; positive control group, 500 μL of dexamethasone at 10 μg/mL, with the final LPS concentration adjusted to 1 μg/mL; and treatment groups(The doses with no obvious cytotoxicity were selected, the drug dose was 2 mg/mL), 500 μL of the drug-containing medium at different concentrations, where the highest safe dose was designated as the high-dose group, followed by two-fold serial dilutions to establish the medium- and low-dose groups, with the final LPS concentration adjusted to 1 μg/mL. After 24 h of incubation, the cell supernatant was collected and centrifuged at 4 °C at 5,000 rpm for 10 min. The resulting supernatant was collected, and the NO content was measured according to the assay kit instructions.

### 2.4 Animal grouping

Male Sprague–Dawley rats of specific pathogen-free grades were obtained from Hunan Silaike Jingda Experimental Animal Co., Ltd. The experimental protocol was approved by the Ethics Committee of Hunan University of Chinese Medicine (Approval No. HNUCM21-2310-28). The rats were housed in the Experimental Animal Center of the Hunan University of Chinese Medicine under controlled environmental conditions: temperature at 22–26 °C, humidity at 60–65%, and a 12-h light–dark cycle, with free access to food and water. The rats were divided into groups by random number table method, *n* = 6 in each group: normal control, LPS, raw carp scale (Cc-LI, 1 g/kg), processed carp scale (Cc-LII, 1 g/kg), and positive control (dexamethasone, 5 mg/kg) groups. The carp scale groups received preventive treatment twice daily for 7 days before injection of LPS, while dexamethasone was administered once, 1 h before injection of LPS. On day 7, the LPS (10 mg/kg) was injected intraperitoneally (14). After 4 h post-injection, blood and organ samples, including blood, heart, lung, liver, and kidney, were collected. The harvested tissues were fixed in 4% paraformaldehyde.

### 2.5 Measurement of serum inflammatory factors TNF-*α*, IL-6, IL-1β, and HMGB1

Blood samples were collected into clean centrifuge tubes and allowed to stand at room temperature for 2 h. The samples were then centrifuged at 3,000 r/min for 20 min, after which the supernatant (serum) was collected. Serum levels of TNF-α, IL-6, IL-1β, and HMGB1 were quantified following the instructions provided in the ELISA kits, the absorbance was measured at 450 nm using a microplate reader.

### 2.6 Measurement of serum oxidative stress indicators MDA and GSH-PX

Blood samples were collected into clean centrifuge tubes and allowed to stand at room temperature for 2 h. The samples were then centrifuged at 3,000 r/min for 20 min, after which the supernatant (serum) was collected. Serum levels of *MDA* and *GSH-PX* were quantified following the instructions provided in the assay kits.

### 2.7 Measurement of liver function indicators (ALT and AST) and kidney function indicators (BUN and Cr)

Blood samples were collected into clean centrifuge tubes and allowed to stand at room temperature for 2 h. The samples were then centrifuged at 3,000 r/min for 20 min, after which the supernatant (serum) was collected. Alanine aminotransferase (ALT), aspartate aminotransferase (AST), blood urea nitrogen (BUN), and serum creatinine (Scr) levels were determined using an automatic biochemical analyzer.

### 2.8 Hematologic system detection

Plasma prothrombin time (PT) was measured using an automatic coagulation analyzer, while platelet count (PLT) in whole blood was evaluated using a hematology analyzer.

### 2.9 HE staining for pathological examination of heart, lung, liver, and kidney tissues

Tissues were rinsed with PBS to remove residual fluids and fixed in 4% paraformaldehyde for 24 h. They were then dehydrated through a graded alcohol and xylene series, embedded in paraffin, and sectioned into 5 μm-thick slices using a Leica RM2235 rotary microtome (Leica Biosystems, Nussloch, Germany). The sections were stained with hematoxylin and eosin (H&E), and images were captured using a microscope.

### 2.10 Statistical analysis

Data were analyzed employing SPSS 25.0 statistical software. Results are expressed as mean ± standard error of the mean (X̅ ± SEM). Comparisons between two groups were conducted using the *t*-test, while multiple group comparisons were conducted with one-way analysis of variance (ANOVA). Statistical significance was defined as *p <* 0.05, high statistical significance as *p* < 0.01, and extreme statistical significance as *p* < 0.001.

## 3 Results

### 3.1 Screening of anti-inflammatory activity *in vitro*

**Figure 1:**
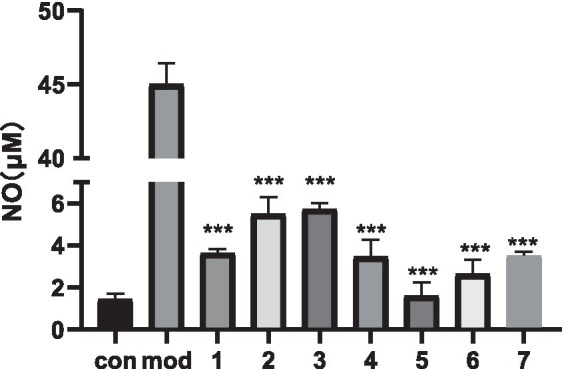
Comparison of anti-inflammatory activities of different processed products; *p < 0.05, **p < 0.01, ***p < 0.001 compared with the model group.

The RAW264.7 cell assay is a well-established method for evaluating anti-inflammatory activity *in vitro*. In the cytotoxicity assay, none of the processed products exhibited cytotoxic effects at the tested concentrations, but all products promoted cell growth to varying degrees. The specific CCK-8 assay data for the seven products are provided in Supplementary Figure S2. Subsequently, the inhibitory effects of the processed products on NO release were evaluated at the same concentrations (Figure 1). The results showed that Method 5 (150 °C, 2.5 min) exhibited the strongest anti-inflammatory activity. Consequently, Method 5 was selected for processing carp scales in subsequent animal experiments.

### 3.2 Analysis of carp scale components

#### 3.2.1 Results of amino acid content determination

**Figure 2:**
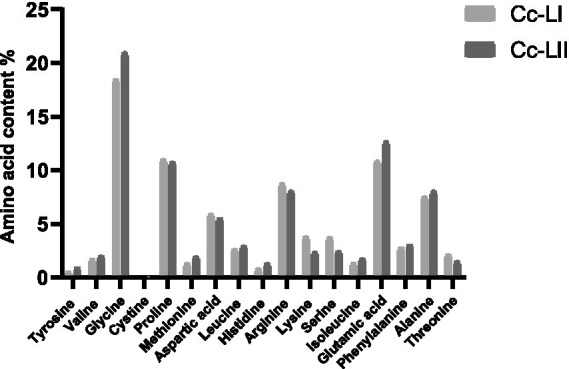
Results of amino acid content determination.

In this study, 17 amino acids were identified in carp scales (Table 1, Figure 2). The total amino acid content in raw carp scales was 82.91%, while that in processed carp scales increased to 85.75%. After processing, the levels of 10 amino acids increased, specifically tyrosine, valine, glycine, methionine, leucine, histidine, isoleucine, glutamic acid, phenylalanine, and alanine. Detailed data are provided in Supplementary material.

**Table 1:** Summary of amino acid content changes.

Test items (%)	Cc-LI		Cc-LII	
	Mean	SD	Mean	SD
Tyrosine	0.55	0.01	0.9	0.01
Valine	1.69	0.01	1.973	0.036
Glycine	18.4	0.1	20.9	0.1
Cystine	0.077	0.006	0.047	0.006
Proline	10.967	0.058	10.7	0.1
Methionine	1.273	0.04	1.937	0.049
Aspartic acid	5.9	0.043	5.48	0.045
Leucine	2.59	0.045	2.917	0.08
Histidine	0.783	0.07	1.273	0.085
Arginine	8.723	0.07	8.017	0.115
Lysine	3.763	0.04	2.357	0.04
Serine	3.7	0.045	2.423	0.058
Isoleucine	1.34	0.02	1.727	0.055
Glutamic acid	10.8	0.1	12.633	0.058
Phenylalanine	2.757	0.006	3.03	0.026
Alanine	7.48	0.052	7.983	0.111
Threonine	2.12	0.026	1.457	0.068

#### 3.2.2 Results of protein content determination

**Figure 3:**
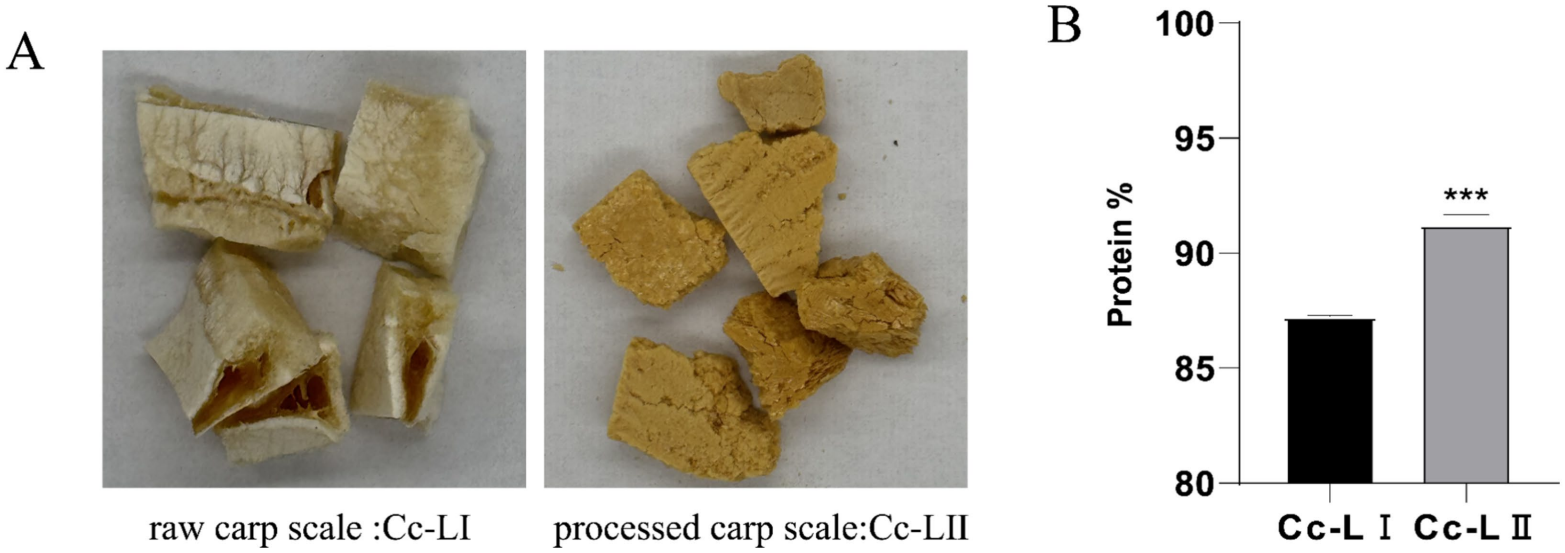
**(A)** Character changes before and after processing. **(B)** Protein content determination, ***p < 0.001 compared with Cc-LI.

The results (Figure 3) of the protein content analysis revealed that the total protein content in raw carp scales was 87.23%, while that in processed carp scales was 91.24% (*p <* 0.001). The higher protein content in processed carp scales suggests an improved nutritional profile.

#### 3.2.3 Results of protein mass spectrometry analysis

**Figure 4:**
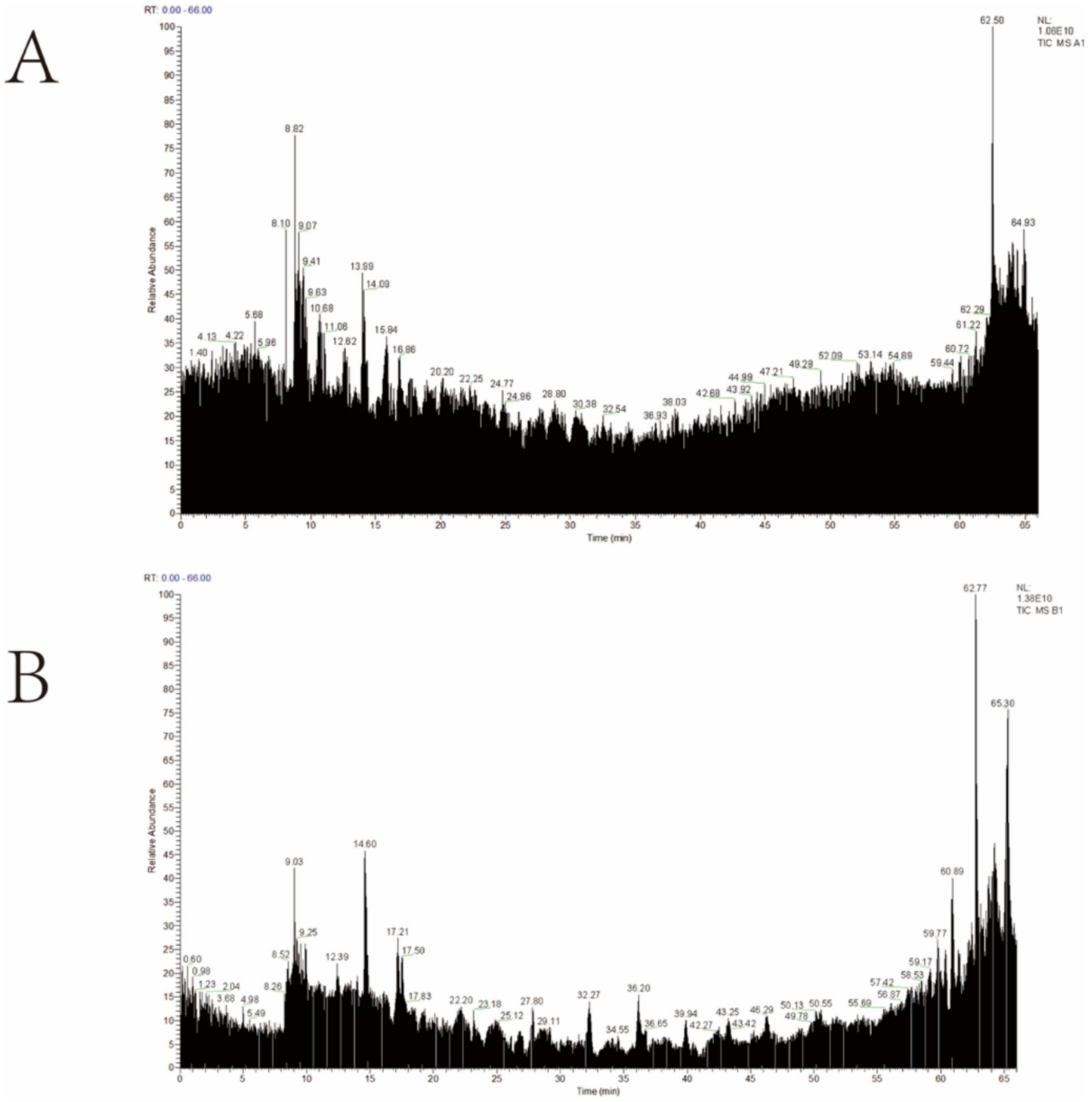
Total ion flow diagram: **(A)** raw carp scale; **(B)** processed carp scale.

Mass spectrometry analysis revealed that (Figure 4), compared to raw carp scales, 10 small molecular peptides were identified in processed carp scales: PGSPGPAGPAGARGQQ, PGAPGA, GPAGAAGPAGN, PAARGPGPSGPSGPKGN, PRRGPSGPAGARGADGN, TSFGRDRTH, AGPAGRHL, YAGG, LYAN, PGPAGASGPAGRGGHL. Details of the secondary mass spectrometry can be found in Supplementary Figure S3. The processed carp scales exhibited superior anti-inflammatory activity *in vivo*.

### 3.3 Detection of inflammatory factors

**Figure 5:**
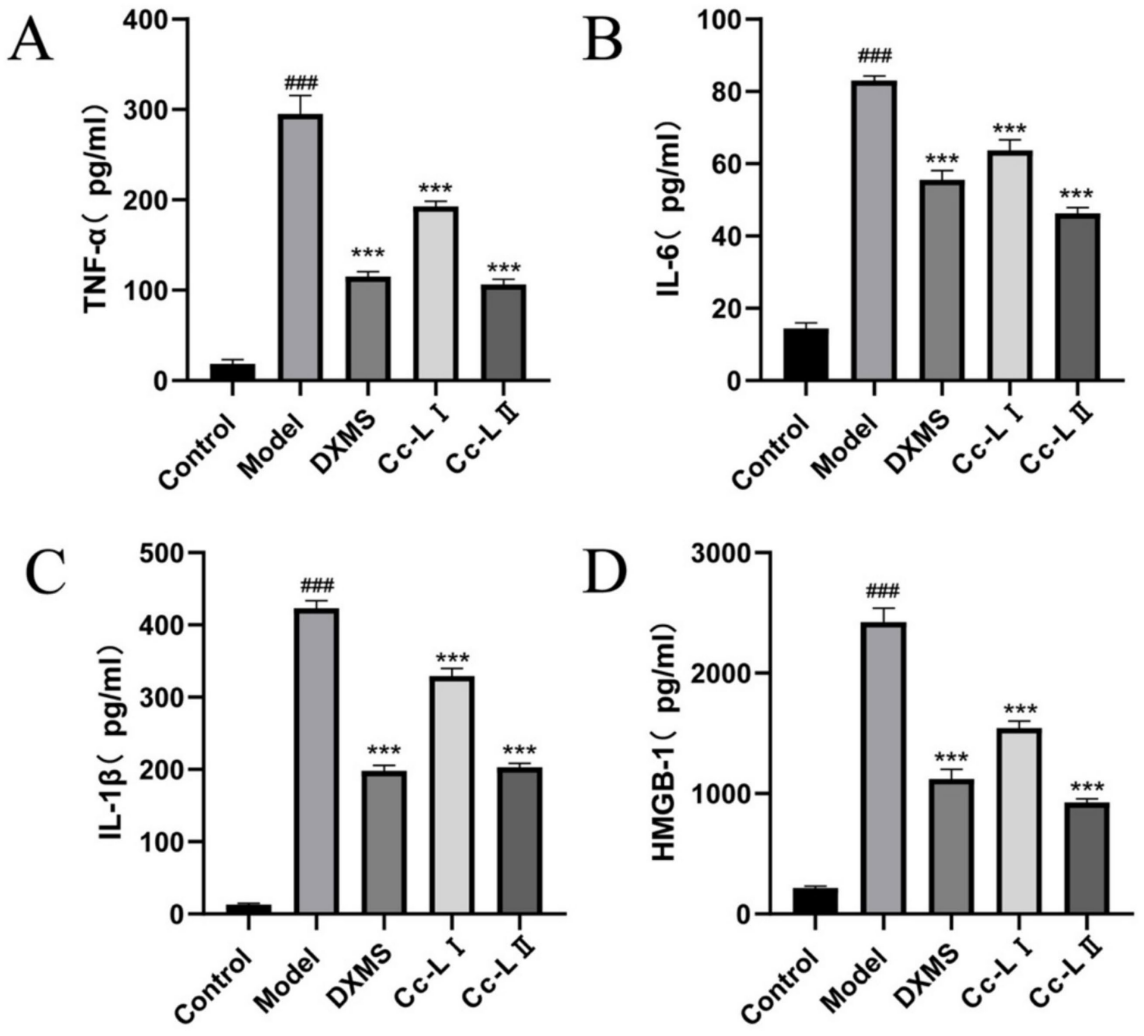
Expression of serum inflammatory factors (mean ± SEM, *n* = 6): **(A)**TNF-α; **(B)** IL-6; **(C)** IL-1β; **(D)** HMGB1; ^###^*p* < 0.001 compared with the control group, *p < 0.05, **p < 0.01, ***p < 0.001 compared with the model group.

ELISA results demonstrated that (Figure 5), compared to the control group, the levels of the four pro-inflammatory factors—TNF-*α*, IL-6, IL-1β, and HMGB1—were significantly elevated in the model group (*p* < 0.01). Compared to the model group, all treatment groups exhibited varying degrees of reduction in these factors, with Cc-L II showing superior efficacy to Cc-L I.

### 3.4 Serum oxidative stress indicators

**Figure 6:**
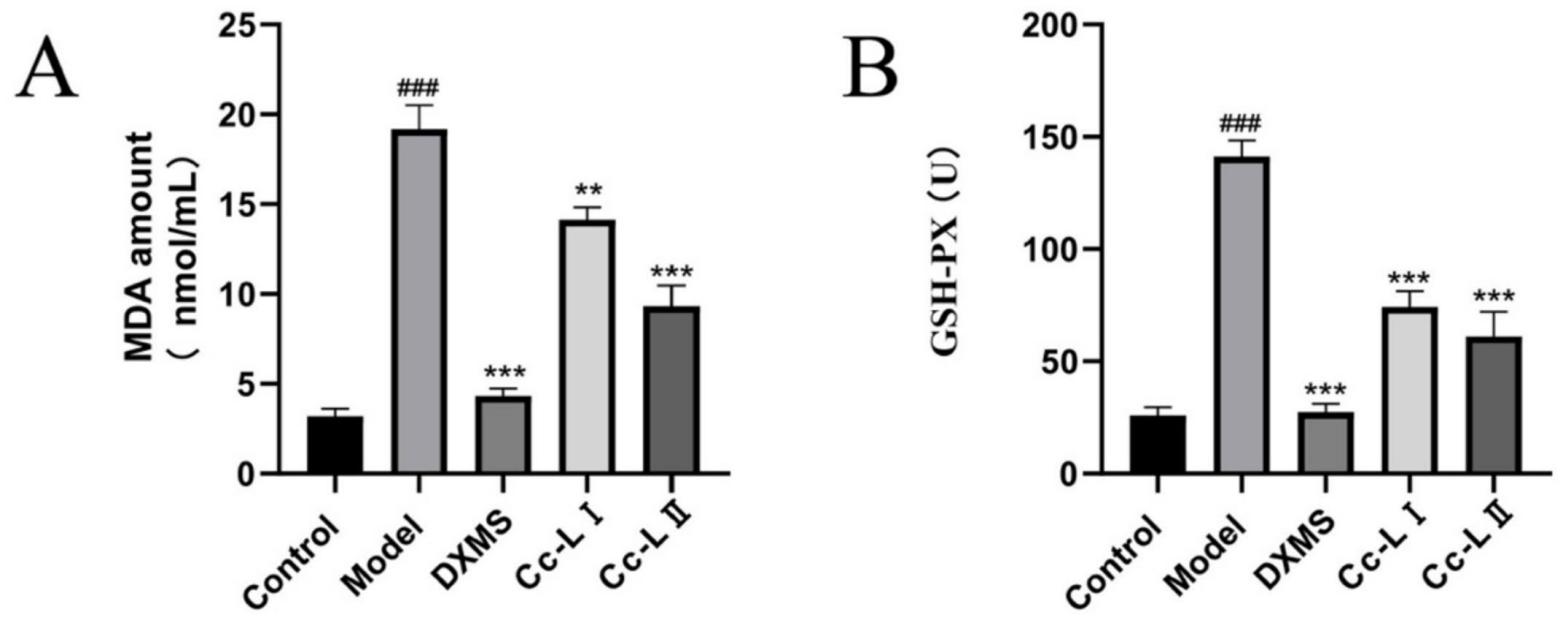
Changes in serum oxidative stress (mean ± SEM, *n* = 6): **(A)** MDA; **(B)** GSH-PX; ^###^*p* < 0.001 compared with the control group, *p < 0.05, **p < 0.01, ***p < 0.001 compared with the model group.

Serum GSH-PX and MDA levels serve as key indicators for evaluating oxidative stress severity. Compared to the control group, the model group exhibited significantly higher GSH-PX and MDA levels (*p* < 0.01). Compared to the model group, all treatment groups exhibited varying degrees of reduction in these indicators, with Cc-L II demonstrating superior efficacy to Cc-L I (Figure 6).

### 3.5 Liver function

**Figure 7:**
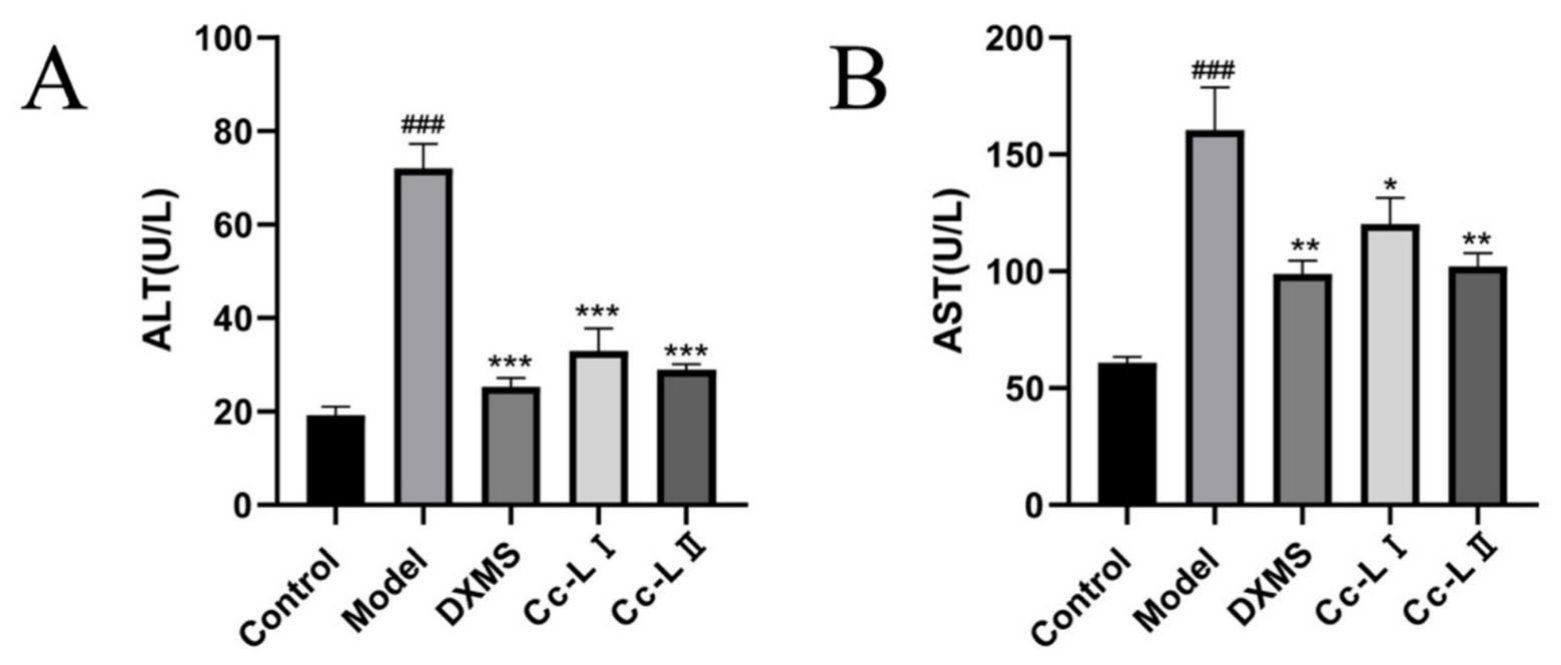
Changes in liver function indices (mean ± SEM, *n* = 6): **(A)** ALT; **(B)** AST; ^###^*p* < 0.001 compared with the control group, *p < 0.05, **p < 0.01, ***p < 0.001 compared with the model group.

Compared to the control group, the model group exhibited significantly higher levels of ALT and AST (*p* < 0.01). Compared to the model group, all treatment groups exhibited varying degrees of reduction in these markers, with Cc-L II demonstrating superior efficacy to Cc-L I (Figure 7).

### 3.6 Kidney function

**Figure 8:**
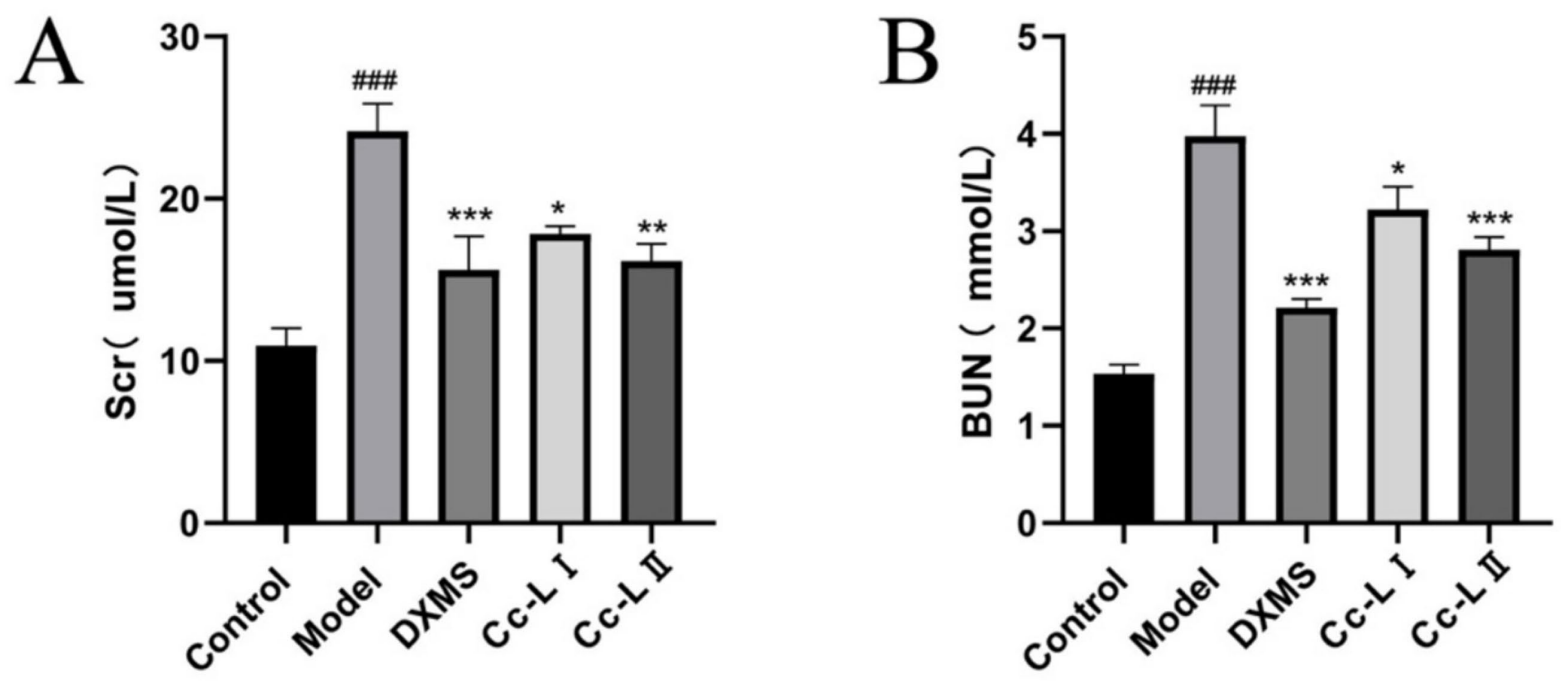
Changes in kidney function indices (mean ± SEM, *n* = 6): **(A)** Scr; **(B)** BUN; ^###^*p* < 0.001 compared with the control group, *p < 0.05, **p < 0.01, ***p < 0.001 compared with the model group.

Compared to the control group, the model group exhibited significantly higher levels of BUN and Scr (*p* < 0.01). Compared to the model group, all treatment groups demonstrated varying degrees of reduction in these markers, with Cc-L II exhibiting superior efficacy to Cc-L I (Figure 8).

### 3.7 Coagulation function analysis

**Figure 9:**
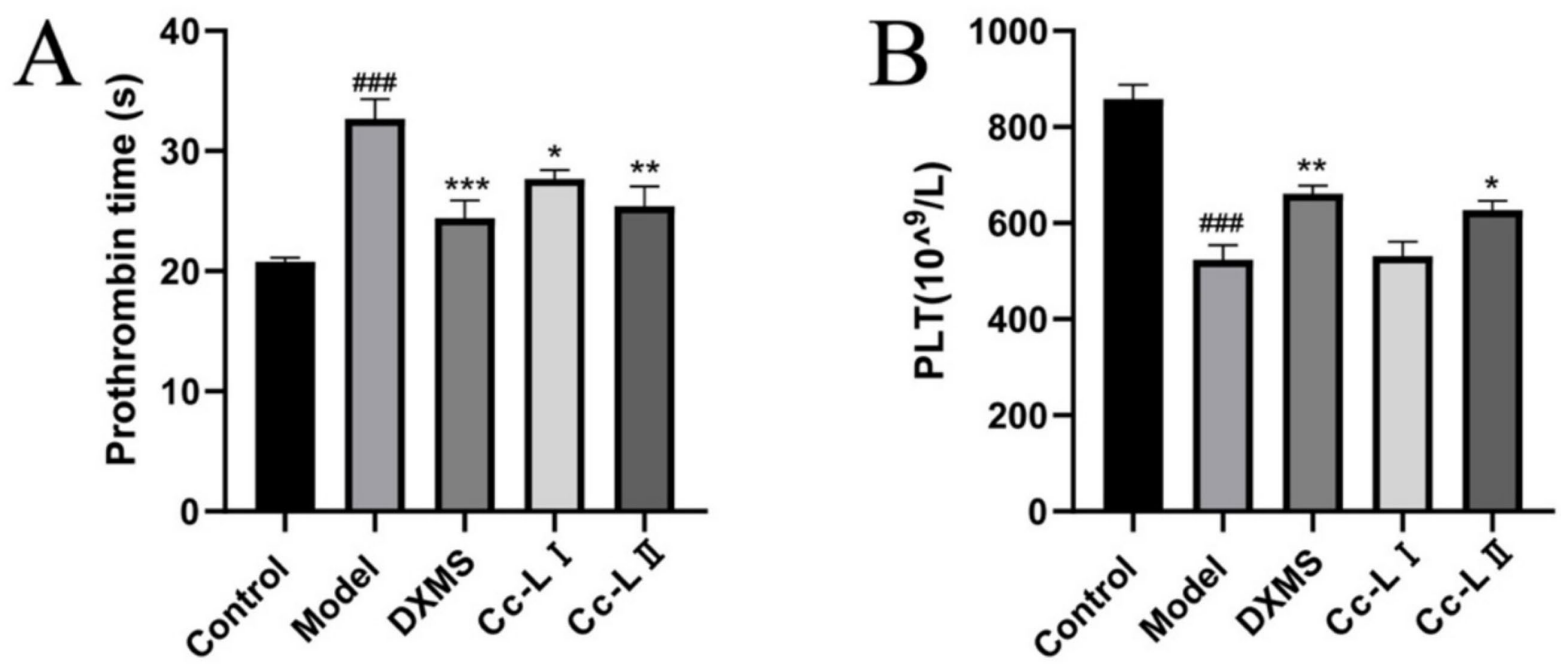
Changes in coagulation function (mean ± SEM, *n* = 6): **(A)** PT; **(B)** PLT; ^###^*p* < 0.001 compared with the control group, *p < 0.05, **p < 0.01, ***p < 0.001 compared with the cmodel group.

Compared to the control group, the model group exhibited significant prolongation of plasma PT (*p* < 0.01). Compared to the model group, all treatment groups exhibited varying degrees of reduction in PT, with Cc-L II demonstrating superior efficacy compared to Cc-L I. Additionally, the model group exhibited significantly reduced PLT counts compared to the control group (*p* < 0.01). Compared to the model group, all treatment groups demonstrated varying degrees of increase in PLT counts, with Cc-L II exhibiting superior efficacy to Cc-L I (Figure 9).

### 3.8 Pathological changes in rat heart, liver, lung, and kidney tissues

#### 3.8.1 Lung tissue (H&E staining)

**Figure 10:**
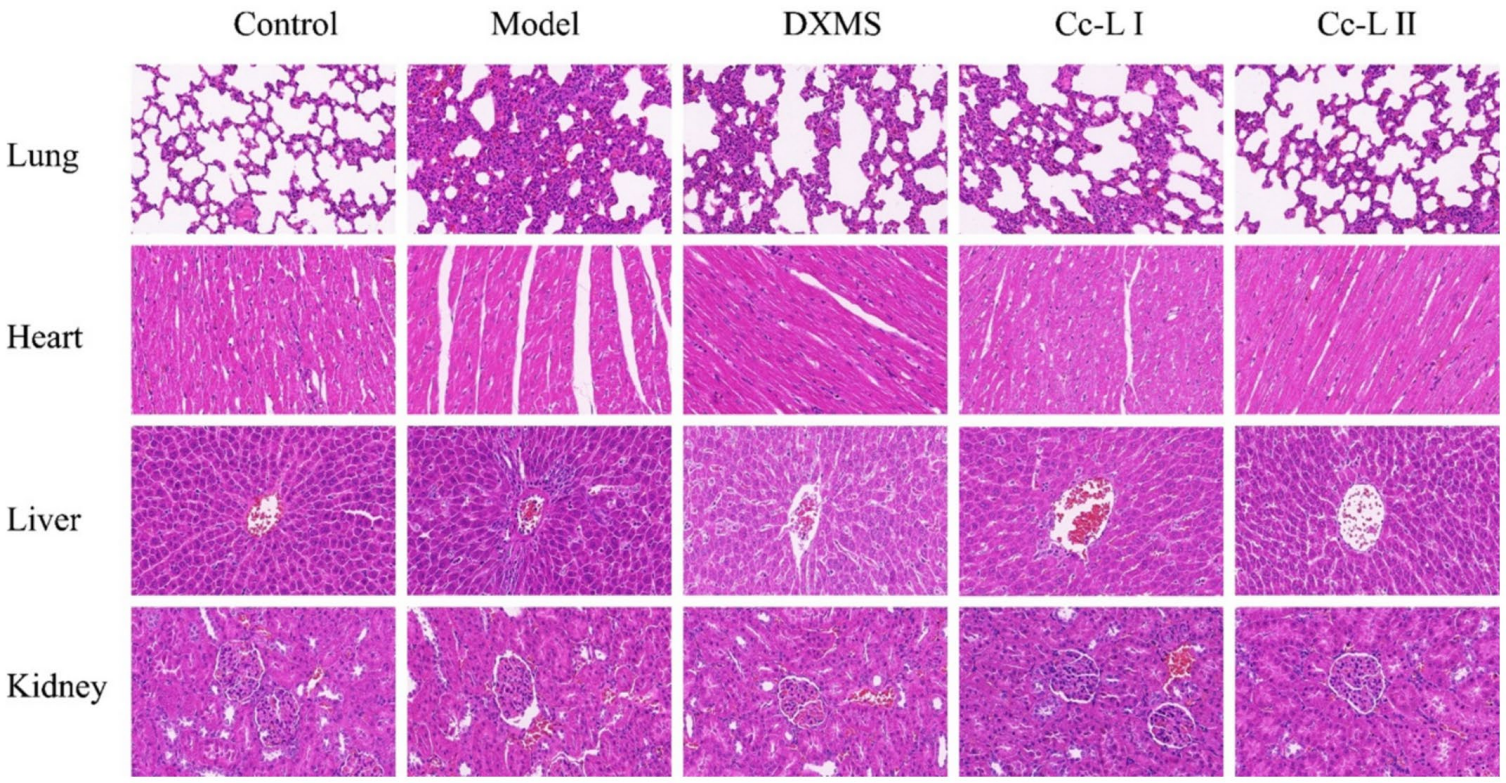
Pathological changes in various organs.

The lung tissue in the control group exhibited normal morphology with a well-defined structure and no significant inflammatory cell infiltration. In the model group, inflammatory cell infiltration and thickening of alveolar septa were observed. In all treatment groups, compared to the model group, inflammatory cell infiltration and alveolar septal thickening were alleviated (Figure 10).

#### 3.8.2 Heart tissue (H&E staining)

The myocardial cells in the control group were arranged orderly, maintaining a normal tissue structure. In the model group, myocardial fibers were disorganized and fragmented, with widened interbundle spaces and pronounced pathological alterations. In all treatment groups, myocardial fiber arrangement remained slightly disordered, but pathological damage was significantly reduced.

#### 3.8.3 Liver tissue (H&E staining)

In the control group, liver cells were radially arranged around the central vein, displaying no significant morphological changes. In the model group, extensive inflammatory cell infiltration was observed around the hepatic sinusoids. In all treatment groups, inflammatory cell infiltration around the hepatic sinusoids was significantly reduced, and pathological damage was alleviated.

#### 3.8.4 Kidney tissue (H&E staining)

The kidney tissue in the control group exhibited an intact structure with no signs of congestion or inflammatory cell infiltration. In the model group, glomeruli appeared shrunken, with noticeable congestion and significant interstitial edema between the renal tubular epithelial cells. In all treatment groups, renal tubular swelling and glomerular damage were significantly reduced.

## 4 Discussion

The development of functional foods derived from aquatic by-products relies critically on the exploration of efficient processing methods. In this study, we demonstrate that applying a traditional sand stir-frying technique (Method 5: 150 °C, 2.5 min) induces significant biochemical alterations in carp scales. Comparative analysis indicates that processed carp scales contain a higher concentration of soluble proteins and free amino acids than the raw material. An increase in amino acid content in food can enhance immune function through various mechanisms, including supporting immune cell proliferation, promoting antibody synthesis, regulating inflammatory responses, strengthening intestinal immunity, bolstering antioxidant defense, facilitating immune signaling, and improving immune aging (15–17). Moreover, LC–MS analysis exclusively detected 10 specific low-molecular-weight peptides (e.g., PGSPGPAGPAGARGQQ, PGAPGA) following thermal treatment. These compositional changes are likely attributed to the thermal modification of the fish scale matrix. Fish scales consist of a rigid structure where type I collagen is mineralized with hydroxyapatite. We propose that the applied heat (150 °C) facilitates the partial unfolding of the collagen triple helix and the disruption of peptide bonds within the mineralized matrix. This physical transformation enhances the release of shorter, more soluble peptide chains and exposes hydrophobic amino acid residues previously buried within the native protein structure (18, 19). These results recapitulate previous findings by Mirzapour-Kouhdasht et al. (20), who reported that reducing the molecular weight of gelatin hydrolysates significantly improves their functional properties. While enzymatic hydrolysis remains a standard and highly effective method for peptide production, our findings suggest that traditional thermal processing offers a chemical-free and cost-efficient alternative. This method may be particularly advantageous for industrial applications where avoiding exogenous enzymes is desired, thereby expanding the methodological toolkit for fish scale valorization.

Understanding the relationship between peptide structure and biological activity is essential for clarifying the potential health benefits of processed carp scales. The newly identified peptides in the processed group, such as PGSPGPAGPAGARGQQ and YAGG, exhibited distinct sequence characteristics. A detailed analysis reveals that these sequences are predominantly rich in Glycine (Gly), Proline (Pro), and Arginine (Arg). As described in research, the unique cyclic structure of Proline contributes to the stability of peptides, potentially enhancing their resistance to gastrointestinal digestion (21, 22). Furthermore, peptides containing the Pro-Gly motif have been suggested to act as hydrogen donors, which may contribute to their ability to scavenge free radicals (23, 24). Notably, the identification of Tyrosine (Tyr)-containing peptides (YAGG, LYAN) in the processed Cc-L Is of particular interest. Je et al. (25) highlighted that the phenolic hydroxyl group of Tyrosine is critical for electron donation, a key mechanism in antioxidant activity. In the context of inflammation, low-molecular-weight antioxidant peptides can help mitigate oxidative stress, which is often an upstream trigger for inflammatory cascades. Additionally, positively charged residues like Arginine (found in PRRGPS…) have been reported to interact with immune cells, potentially modulating their response (26). While the individual contribution of each peptide requires further isolation and specific testing, the synergistic presence of these bioactive sequences likely underlies the enhanced overall activity observed in the processed material compared to the raw scales.

To evaluate the physiological relevance of these compositional changes, we utilized an LPS-induced rats sepsis model, which mimics systemic inflammation and multiorgan stress (14). Our *in vivo* data show that treatment with processed carp scales was associated with a significant reduction in serum pro-inflammatory cytokines, including TNF-*α*, IL-6, IL-1β, and HMGB1 (27, 28). The reduction of these markers mirrors effects reported for general therapeutic strategies for sepsis, which aim to control the “cytokine storm” to prevent tissue damage (29). The observed effects are similar to those of other marine-derived anti-inflammatory agents, indicating that processed carp scales possess comparable bioactive potential. The mechanism appears to involve a dual action of immune modulation and improvement of oxidative status. Sepsis is characterized by a vicious cycle of inflammation and oxidative damage (11). In our study, processed carp scales not only lowered inflammatory cytokines but also improved oxidative status—evidenced by decreased MDA and increased GSH levels. This restoration of the redox status likely contributed to the preservation of organ function, as confirmed by the lower levels of liver and kidney injury markers (ALT, AST, BUN) and reduced neutrophil infiltration in histological sections (30, 31). The superior efficacy of processed scales over raw scales is attributed to the improved bioavailability of the low-molecular-weight peptides generated during processing, allowing for more effective systemic distribution.

The sustainable utilization of aquaculture by-products is a significant objective in modern food science. Previous successful applications have incorporated fish collagen hydrolysates into beverages primarily for skin health and anti-aging purposes (32, 33). The present study extends this scope by providing preliminary evidence that processed carp scales may also support immune health and manage systemic inflammation. By demonstrating that a straightforward, eco-friendly processing method can enhance the nutritional and functional value of carp scales, this work supports the “waste-to-wealth” concept. The processed carp scale powder, characterized by its naturally derived peptides and amino acids, represents a promising candidate for inclusion in functional foods or dietary supplements. However, it is important to note that while the results are encouraging, the precise molecular targets and clinical translatability warrant further investigation. Future studies should focus on the purification of specific peptides and the elucidation of detailed signaling pathways to fully validate their potential as therapeutic functional foods.

## 5 Conclusion

In conclusion, this study demonstrates that traditional thermal processing (150 °C, 2.5 min) effectively changes the nutritional profile of carp scales, significantly increasing total protein content and generating novel low-molecular-weight peptides. *In vivo* experiments confirmed that these compositional changes translate into enhanced anti-inflammatory and organ-protective efficacy against sepsis compared to raw scales. Importantly, these findings highlight a sustainable strategy for valorizing aquaculture by-products, transforming discarded waste into high-value functional food ingredients. While promising, further research is warranted to elucidate the precise molecular mechanisms of individual peptides and to validate their clinical efficacy in future applications.

## Data Availability

The original contributions presented in the study are included in the article/Supplementary material, further inquiries can be directed to the corresponding author.

## References

[1] Kaushik N Falch E Slizyte R Kumari A Khushboo Hjellnes V . Valorization of fish processing by-products for protein hydrolysate recovery: opportunities, challenges and regulatory issues. Food Chem. (2024) 459:140244. doi: 10.1016/j.foodchem.2024.140244 38991448

[2] Singh H Sharma R Gupta A Joshi S Dar BN Singh B . Characterization of jackfruit seed enriched pasta: product-functionality profile, secondary protein structures, bioactive composition and molecular morphology. Qual Assur Saf Crop Foods. (2023) 15:11–9. doi: 10.15586/qas.v15i2.1217

[3] Nirmal NP Santivarangkna C Rajput MS Benjakul S Maqsood S. Valorization of fish byproducts: sources to end-product applications of bioactive protein hydrolysate. Compr Rev Food Sci Food Saf. (2022) 21:1803–42. doi: 10.1111/1541-4337.12917, 35150206

[4] Sivaraman K Shanthi C. Purified fish skin collagen hydrolysate attenuates TNF-α induced barrier dysfunction *in-vitro* and DSS induced colitis in-vivo model. Int J Biol Macromol. (2022) 222:448–61. doi: 10.1016/j.ijbiomac.2022.09.122, 36116587

[5] Cho W Park J Kim J Lee M Park SJ Kim KS . Low-molecular-weight fish collagen peptide (valine-Glycine-proline-Hydroxyproline-Glycine-proline-alanine-Glycine) prevents osteoarthritis symptoms in chondrocytes and monoiodoacetate-injected rats. Mar Drugs. (2023) 21:608. doi: 10.3390/md21120608 38132929 PMC10744650

[6] Kim HM Jin BR Lee JS Jo EH Park MC An HJ. Anti-atopic dermatitis effect of fish collagen on house dust mite-induced mice and HaCaT keratinocytes. Sci Rep. (2023) 13:14888. doi: 10.1038/s41598-023-41831-w, 37689763 PMC10492863

[7] Zu XY Huang YQ Zhao YJ Xiong GQ Liao T Li HL. Peptide extraction from silver carp scales via enzymatic hydrolysis and membrane filtration. Ital J Food Sci. (2023) 35:44–53. doi: 10.15586/ijfs.v35i2.2248

[8] Chai HJ Wu CJ Yang SH Li TL Pan BS. Peptides from hydrolysate of lantern fish (*Benthosema pterotum*) proved neuroprotective *in vitro* and *in vivo*. J Funct Foods. (2016) 24:438–49. doi: 10.1016/j.jff.2016.04.009

[9] Miao B Zheng J Zheng G Tian X Zhang W Yuan F . Using collagen peptides from the skin of monkfish (*Lophius litulon*) to ameliorate kidney damage in high-fat diet fed mice by regulating the Nrf2 pathway and NLRP3 signaling. Front Nutr. (2022) 9:798708. doi: 10.3389/fnut.2022.798708 35223948 PMC8866304

[10] Singer M Deutschman CS Seymour CW Shankar-Hari M Annane D Bauer M . The third international consensus definitions for Sepsis and septic shock (Sepsis-3). JAMA. (2016) 315:801–10. doi: 10.1001/jama.2016.0287, 26903338 PMC4968574

[11] Wang G Li X Zhang L Elgaili Abdalla A Teng T Li Y. Crosstalk between dendritic cells and immune modulatory agents against sepsis. Genes Basel. (2020) 11:323. doi: 10.3390/genes11030323 32197507 PMC7140865

[12] Park JH Lee SI Kwon WS Cho SB Kim IH. Health benefits of co-supplementing mealworm protein hydrolysate and cranberry fruit extract. Ital J Food Sci. (2023) 35:1–9. doi: 10.15586/ijfs.v35i1.2264

[13] Lei Y Xu D Wang Y Guo S Li L Luo M . Triterpenoid saponins from *Rubus setchuenensis* roots and their anti-inflammatory activities *in vitro*. Phytochemistry. (2025) 233:114403. doi: 10.1016/j.phytochem.2025.114403 39832633

[14] Temiz-Resitoglu M Kucukkavruk SP Guden DS Cecen P Sari AN Tunctan B . Activation of mTOR/IkappaB-alpha/NF-kappaB pathway contributes to LPS-induced hypotension and inflammation in rats. Eur J Pharmacol. (2017) 802:7–19. doi: 10.1016/j.ejphar.2017.02.034, 28228357

[15] Cruzat V Macedo Rogero M Keane KN Curi R Newsholme P. Glutamine: metabolism and immune function, supplementation and clinical translation. Nutrients. (2018) 10:1564. doi: 10.3390/nu10111564 30360490 PMC6266414

[16] Jongkees BJ Hommel B Kühn S Colzato LS. Effect of tyrosine supplementation on clinical and healthy populations under stress or cognitive demands--a review. J Psychiatr Res. (2015) 70:50–7. doi: 10.1016/j.jpsychires.2015.08.014, 26424423

[17] Negro M Giardina S Marzani B Marzatico F. Branched-chain amino acid supplementation does not enhance athletic performance but affects muscle recovery and the immune system. J Sports Med Phys Fitness. (2008) 48:347–51. 18974721

[18] Hussain S Rahman A Borah P Sen A Bharalee R Chabukdhara M . Eco-friendly advancements through fish waste: a review of therapeutic and industrial innovations. Comb Chem High Throughput Screen. (2025) e13862073372954. doi: 10.2174/0113862073372954250408181058 40277112

[19] Jeyachandran S Aman M. Valorisation of fish scales and bones: a sustainable source of bioactive proteins and collagen for nutraceuticals. Bioresour Bioprocess. (2025) 12:141. doi: 10.1186/s40643-025-00970-w, 41335283 PMC12675900

[20] Mirzapour-Kouhdasht A Moosavi-Nasab M Kim YM Eun JB. Antioxidant mechanism, antibacterial activity, and functional characterization of peptide fractions obtained from barred mackerel gelatin with a focus on application in carbonated beverages. Food Chem. (2021) 342:128339. doi: 10.1016/j.foodchem.2020.128339, 33069523

[21] Udenigwe CC Aluko RE. Food protein-derived bioactive peptides: production, processing, and potential health benefits. J Food Sci. (2012) 77:R11–24. doi: 10.1111/j.1750-3841.2011.02455.x, 22260122

[22] Zu XY Zhao YJ Fu SM Liao T Li HL Xiong GQ. Physicochemical properties and biological activities of silver carp scale peptide and its nanofiltration fractions. Front Nutr. (2021) 8:812443. doi: 10.3389/fnut.2021.812443 35059429 PMC8765580

[23] Sila A Bougatef A. Antioxidant peptides from marine by-products: isolation, identification and application in food systems. A review. J Funct Foods. (2016) 21:10–26. doi: 10.1016/j.jff.2015.11.007

[24] Zou TB He TP Li HB Tang HW Xia EQ. The structure-activity relationship of the antioxidant peptides from natural proteins. Molecules. (2016) 21:72. doi: 10.3390/molecules21010072, 26771594 PMC6273900

[25] Je JY Park PJ Kwon JY Kim SK. A novel angiotensin I converting enzyme inhibitory peptide from Alaska pollack (*Theragra chalcogramma*) frame protein hydrolysate. J Agric Food Chem. (2004) 52:7842–5. doi: 10.1021/jf0494027, 15612765

[26] Admassu H Gasmalla MAA Yang R Zhao W. Bioactive peptides derived from seaweed protein and their health benefits: antihypertensive, antioxidant, and antidiabetic properties. J Food Sci. (2018) 83:6–16. doi: 10.1111/1750-3841.14011, 29227526

[27] Deng M Scott MJ Fan J Billiar TR. Location is the key to function: HMGB1 in sepsis and trauma-induced inflammation. J Leukoc Biol. (2019) 106:161–9. doi: 10.1002/JLB.3MIR1218-497R, 30946496 PMC6597316

[28] Kumar V. Correction to: Immunometabolism: another road to sepsis and its therapeutic targeting. Inflammation. (2019) 42:789. doi: 10.1007/s10753-019-00970-x, 30737663

[29] Tsukamoto H Takeuchi S Kubota K Kobayashi Y Kozakai S Ukai I . Lipopolysaccharide (LPS)-binding protein stimulates CD14-dependent toll-like receptor 4 internalization and LPS-induced TBK1-IKKϵ-IRF3 axis activation. J Biol Chem. (2018) 293:10186–201. doi: 10.1074/jbc.M117.796631, 29760187 PMC6028956

[30] Hwang JS Kim KH Park J Kim SM Cho H Lee Y . Glucosamine improves survival in a mouse model of sepsis and attenuates sepsis-induced lung injury and inflammation. J Biol Chem. (2019) 294:608–22. doi: 10.1074/jbc.RA118.004638, 30455348 PMC6333887

[31] Kounatidis D Vallianou NG Psallida S Panagopoulos F Margellou E Tsilingiris D . Sepsis-associated acute kidney injury: where are we now? Medicina. (2024) 60:19. doi: 10.3390/medicina60030434 PMC10971830 38541160

[32] Bilek SE Bayram SK. Fruit juice drink production containing hydrolyzed collagen. J Funct Foods. (2015) 14:562–9. doi: 10.1016/j.jff.2015.02.024

[33] Tanaka M Koyama Y Nomura Y. Effects of collagen peptide ingestion on UV-B-induced skin damage. Biosci Biotechnol Biochem. (2009) 73:930–2. doi: 10.1271/bbb.80649, 19352014

