# Supplementary material for: Processed carp scales as a functional food for sepsis protection: enhancing amino acid content and bioactive peptide profiles
Source: Front Nutr. 2026 Jan 26;13:1723040. doi: 10.3389/fnut.2026.1723040 (PMC12883412; doi:10.3389/fnut.2026.1723040)
Supplement: Supplementary file 1 [file Supplementary_file_1.docx]

**Fig 1**. **Fig 1**. Comparison of anti-inflammatory activities of different processed products; **P* < 0.05, ***P* < 0.01, ****P* < 0.001 compared with the model group.

**Fig 2 A**. Character changes before and after processing; B: Protein content determination, ***P < 0.001 compared with Cc-LⅠ.

**Fig 3**. Results of amino acid content determination.

**Fig 4**. Total ion flow diagram: (A) Raw carp scale; (B) processed carp scale.

**Fig 5**. Expression of serum inflammatory factors (mean ± SEM, n = 6), ^###^P < 0.001 compared with the control group, *P < 0.05, **P < 0.01, ***P < 0.001 compared with the model group.

**Fig 6**. Changes in serum oxidative stress (mean ± SEM, n = 6), ^###^P < 0.001 compared with the control group, *P < 0.05, **P < 0.01, ***P < 0.001 compared with the model group.

**Fig 7**. Changes in liver function indices (mean ± SEM, n = 6), ^###^P < 0.001 compared with the control group, *P < 0.05, **P < 0.01, ***P < 0.001 compared with the model group.

**Fig 8**. Changes in kidney function indices (mean ± SEM, n = 6), ^###^P < 0.001 compared with the control group, *P < 0.05, **P < 0.01, ***P < 0.001 compared with the model group.

**Fig 9**. Changes in coagulation function (mean ± SEM, n = 6), ^###^P < 0.001 compared with the Control group, *P < 0.05, **P < 0.01, ***P < 0.001 compared with the model group.

**Fig 10.** Pathological changes in various organs.
